# Intragenic suppressors of *unc-104* ( *e1265* ) identify potential roles of the conserved stalk region

**DOI:** 10.17912/micropub.biology.000539

**Published:** 2022-04-06

**Authors:** Dana T Byrd, Julie M Pearlman, Yishi Jin

**Affiliations:** 1 Department of Neurobiology, School of Biological Sciences, University of California San Diego, CA, USA; 2 Department of MCD Biology, Sinsheimer Laboratories, University of California Santa Cruz, Santa Cruz, CA, USA

## Abstract

UNC-104 and its mammalian ortholog, KIF1A, are microtubule motor proteins required for moving synaptic vesicle precursors from neuronal cell bodies to presynaptic sites. These motor proteins consist of N-terminal motor domain, followed by a neck region, three coiled-coil domains and a FHA domain, and a C-terminal PH domain. Between the coiled-coil 3 and the PH domain is a large uncharacterized region called stalk. In *C. elegans* *unc-104* ( *e1265* ), a partial loss of function mutant, synaptic vesicles are retained in the cell body and absent from presynaptic sites. *unc-104* ( *e1265* ) contains amino acid substitution D1497N in the PH domain and the mutant proteins show reduced PI(4,5)P(2) binding. Through genetic suppressor screening, we identified amino acid substitutions in a conserved region of the stalk that cause intragenic suppression of *unc-104* ( *e1265* ). Currently, little is known about the functions of the stalk region. Our findings imply potential compensatory or antagonistic interaction between the stalk region and the cargo binding PH domain.

**Figure 1. Amino acid substitutions in the UNC-104 stalk suppress PH domain-disrupting unc-104 ( e1265 ):**
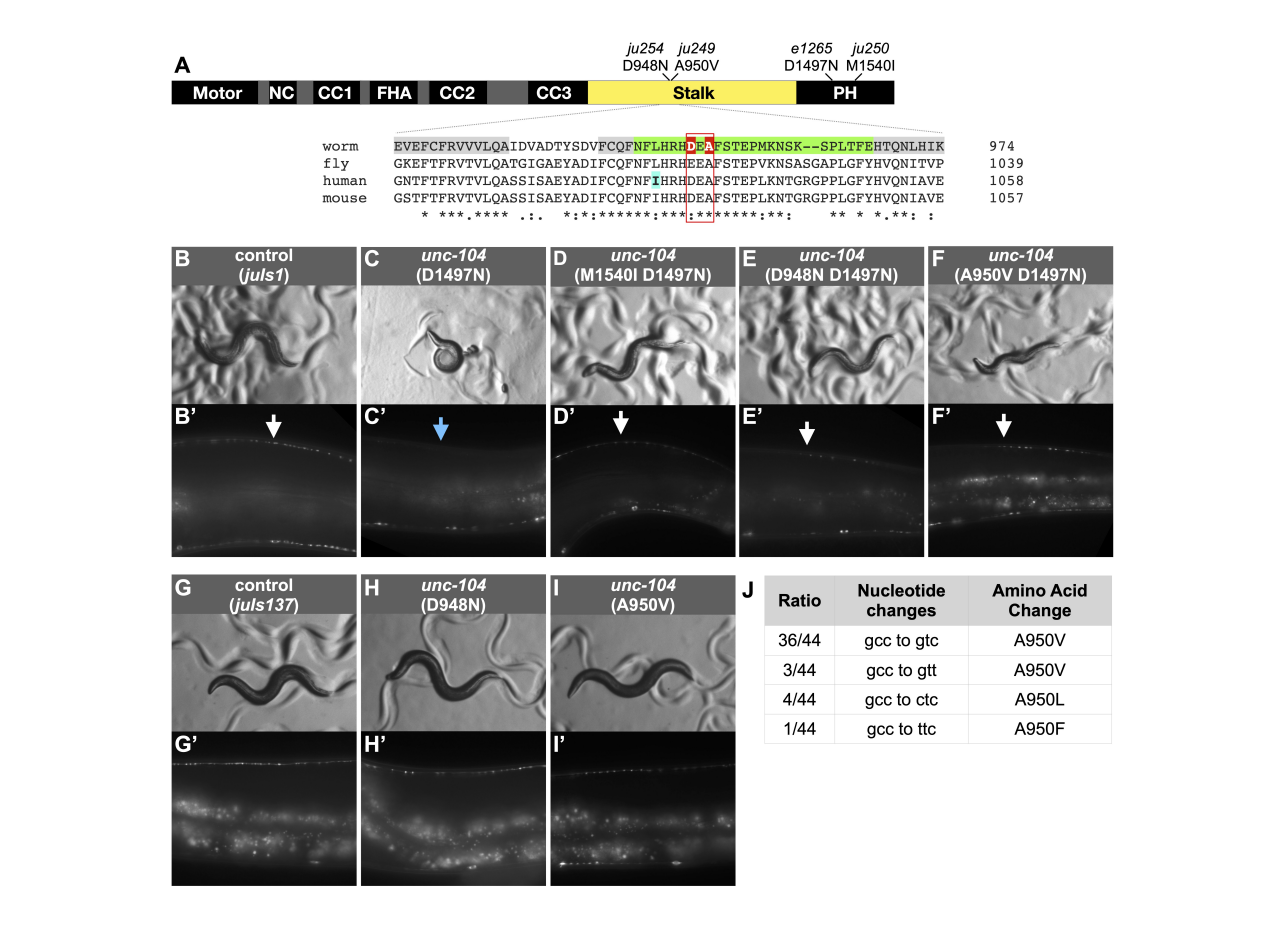
Protein schematic showing domain organization with amino acid changes and alignment of part of the Stalk region. Grey highlighted amino acids in alignment are predicted beta strands in Alpha Fold structure. Green highlighted amino acids in alignment compose conserved loop containing the suppressor positions D948 and A950 (red highlight). SPG30 variant position I1026 denoted with cyan highlight. (B-F) Bright field images showing movement tracks of wild type ( *juIs1* ) (B), *unc-104* ( *e1265* ) (encoding D1497N) (C), and suppressors *unc-104* ( *ju250 e1265* ) (encoding M1540I D1497N) (D), *unc-104* ( *ju254 e1265* ) (encoding D948N D1497N) (E), and *unc-104* ( *ju249 e1265* ) (encoding A950V D1497N) (F). (B’-F’) Expression of the P *unc-25* -SNB-1::GFP( *juIs1* ) synaptic vesicle marker along the dorsal and ventral nerve cords in wild type (B’), *unc-104* ( *e1265* ) (encoding D1497N) (C”), and suppressors (D’-F’). (G-I) Bright field images showing movement tracks of wild type ( *juIs137* ) (G), *unc-104* ( *ju1894* ) (encoding D948N) (H), and *unc-104* ( *ju1895* ) (encoding A950V) (I). (G’-I’) Expression of the P *flp-13* -SNB-1::GFP( *juIs137* ) synaptic vesicle marker along the dorsal and ventral processes of DD motor neurons. (J) Chart showing sequencing results of *ju249* CRISPR rescue of *unc-104* ( *e1265* ). Ratio is number of F1 animals with indicated codon change out of the 44 total suppressed animals that were sequenced.

## Description

*Results and Discussion*

The conserved UNC-104/KIF1A microtubule motor proteins are dedicated to transport presynaptic vesicles from *C. elegans* to human. They consist of N-terminal motor, neck, coiled coil and FHA domains and a C-terminal PH domain separated by a conserved, but largely uncharacterized, stalk (Figure 1A). *unc-104(e1265)* is a widely used partial loss of function mutant, and contains amino acid substitution D1497N in the PH domain (Kumar *et al.* 2010). *unc-104(e1265)* animals are strong coilers (Figure 1C), and synaptic vesicle proteins are trapped in neuronal soma and nearly absent in axons (Figure 1C’; Hall and Hedgecock 1991; Otsuka *et al.* 1991). We previously carried out a suppressor screen of *unc-104(e1265)* for genes involved in synaptic vesicle transport and reported one extragenic suppressor *ju146* to be an allele of *unc-16* , which encodes the ortholog of mammalian JIP3 (Byrd *et al.* 2001). Here we report three intragenic suppressors of *unc-104(e1265)* . One, represented by *ju250* , causes a compensatory M1540I change within the PH domain, identical to a previous report (Kumar *et al.* 2010).

The remaining two intragenic suppressors, represented by *ju254* and *ju249* , cause D948N and A950V, respectively, in the stalk (Figure 1A). Animals of *unc-104(ju254 ju1265)* or *unc-104(ju249 ju1265)* have improved movement, compared to *unc-104(e1265),* and localization of a synaptic vesicle marker is seen in presynaptic regions (Figure 1D-F and D’-F’). To confirm the causative changes, we used CRISPR/Cas9 editing to recreate *ju254* and *ju249* lesions in *unc-104* ( *e1265* ). CRISPR edited *unc-104* ( *e1265* ) animals were identified by improved movement and verified by sequencing, consistent with D948N and A950V indeed being the amino acid substitutions that cause suppression of UNC-104(D1497N). Similar CRISPR editing in a wild type background generated *unc-104* ( *ju1894* ), which contains the *ju254* change D948N only, and *unc-104* ( *ju1895* ), which contains the *ju249* change A950V only *,* neither of which display noticeable changes in movement or localization of a synaptic vesicle marker, compared to controls (Figure 1G-I and G’-I’). Interestingly, while using CRISPR to confirm that A950V (present in *ju249* ) suppresses *unc-104(e1265)* , we also isolated several well-moving animals with codon changes resulting in A950L and A950S (Figure 1J), suggesting that A950 is a critical residue that may influence cargo binding or stability of UNC-104(D1497N). Additionally, we found the sgRNA and repair template (methods) used for editing the A950V nucleotide changes to be highly efficient (Figure 1J), well suited to be used as a co-CRISPR tool when current widely used co-CRISPR markers *dpy-10* or *unc-58* are not desired.

UNC-104(D1497N) motor proteins (as in *unc-104* ( *e1265* )) have reduced PI(4,5)P(2) binding and likely reduced synaptic vesicle binding, resulting in a cargo-dependent inhibition or degradation of UNC-104 (Kumar et al. 2010). We hypothesize that D948N and A950V intramolecular alterations may block the cargo-dependent inhibition, allowing UNC-104(D1497N) to move even with reduced cargo binding. One of the KIF1A variants linked to human hereditary spastic paraplegia (SPG30), p.I1026T (Figure 1A), lies in this same uncharacterized, but highly conserved region within the human UNC-104 ortholog, KIF1A (Citterio *et al. 2015).* Modeling this human variant in *C. elegans* may offer insight into how the function of SPG30 motor protein may be altered.

## Methods

Strains and Genetics.

*C. elegans* strains were grown on NGM plates as described (Brenner, 1974). Unc-104 suppressors were isolated from *unc-104* ( *e1265* ); *juIs1* animals treated with ethyl methanesulfonate as described previously (Byrd *et al.* 2001). During outcrossing, *ju249* , *ju250* , and *ju254* were each found to be linked to *unc-104* ( *e1265* ), we first verified the presence of *e1265* by sequencing. While all contained the *e1265* change G4489A in coding sequence C52E12.2a (encoding D1497N), *ju250* contained an additional nucleotide change, G4620A (encoding M1540I). The remaining exon regions of *unc-104* were sequenced, revealing nucleotide changes C2849T in *ju249* (encoding A950V) and G2842A in *ju254* (encoding D948N).

CRISPR/Cas9 Genome Editing

To verify causative nucleotide changes identified in *ju249* and *ju254,* we designed a crRNA for *unc-104* (Integrated DNA Technologies) toward a site approximately 9 and 16 nucleotides away from the desired edits, respectively. We co-injected into *unc-104* ( *e1265* ); *juIs1* worms with crRNA, purified Cas9 (28 μM, Macrolabs, University of California, Berkeley) plus either a repair oligonucleotide template for *C2849t (ju249* ) or G2842a ( *ju254)* (Integrated DNA Technologies - see sequences below). F1 worms were selected for the non-Unc phenotype and then sequenced for *unc-104* genomic edits. While additional edits to simplify screening were designed in the repair templates, not all edits were isolated in each suppressed animal, but every suppressed animal had codon changes at A950 or D948.

*unc-104* ( *ju1894* ) *juIs137* and *unc-104* ( *ju1895* ) *juIs137* were generated using the *unc-58* co-CRISPR method (Paix *et al.* 2017). *juIs137* animals were injected with the *unc-104* crRNA, *unc-58* crRNA, Cas9 protein, and repair oligonucleotide templates for both *unc-104* and *unc-58* . F1 animals were selected based on the Unc-58 phenotype and sequenced for *unc-104* genomic edits. Non-Unc F2 animals were then selected and outcrossed 3x with N2.

## Reagents

crRNA sequence

*unc-104* : CCATGATGAAGCCTTCTCAA

(Unspliced C5212.2a.1 nucleotides 12,156-12,175)

Repair oligo sequences

*For recreating ju249* :

 CGTGGCAGACACATATTCTGATGTTTTCTGTCAATTCAAgtattgtttaattAaaaacatttccaattgaagaattttattttcagTTTCTTGCACCGCCATGATGAAGtCTTCTCAACaGAGCCAATGAAAAACTCAAAATCTCCATTAACATTCGAAC

[Unspliced C52E12.2a.1 nucleotides 12,058-12,217 with the following changes: t12,110a to destroy DraI site, c12,167t ( *ju249* ), and g12,177a (ACG to ACA still Thr, but destroys PAM)]

For recreating *ju254* :

taaaacatttccaattgaagaattttattttcagTTTCTTGCACCGCCATaATGAAGCtTTCTCAACaGAGCCAATGAAAAACTCAAAATCTCCATTAACATTCGAACACACCCAAAA

[Unspliced C52E12.2a.1 nucleotides 12,110-12,227 with the following changes: g12,160a ( *ju254* ), c12,168t (GCC to GCT still Ala, but adds HindIII), g12,177a (ACG to ACA still Thr, but destroys PAM)]

Strain list

**Table:** 

**Strain**	**Genotype**	**Source**
CZ333	*juIs1(* P *unc-25* -SNB-1::GFP)	Hallam and Jin, 1998
CZ4819	*unc-104(e1265); juIs1*	This study
CZ2165	*unc-104(ju249 e1265); juIs1*	This study
CZ2166	*unc-104(ju250 e1265); juIs1*	This study
CZ2170	*unc-104(ju254 e1265); juIs1*	This study
CZ2060	*juIs137(* P *flp-13* -SNB-1::GFP)	Sakaguchi-Nakashima *et al.* 2007
CZ29059	*unc-104(ju1894) juIs137*	This study
CZ29060	*unc-104(ju1895) juIs137*	This study

## References

[R1] Brenner S (1974). The genetics of Caenorhabditis elegans.. Genetics.

[R2] Byrd DT, Kawasaki M, Walcoff M, Hisamoto N, Matsumoto K, Jin Y (2001). UNC-16, a JNK-signaling scaffold protein, regulates vesicle transport in C. elegans.. Neuron.

[R3] Citterio A, Arnoldi A, Panzeri E, Merlini L, D'Angelo MG, Musumeci O, Toscano A, Bondi A, Martinuzzi A, Bresolin N, Bassi MT (2015). Variants in KIF1A gene in dominant and sporadic forms of hereditary spastic paraparesis.. J Neurol.

[R4] Hall DH, Hedgecock EM (1991). Kinesin-related gene unc-104 is required for axonal transport of synaptic vesicles in C. elegans.. Cell.

[R5] Hallam SJ, Jin Y (1998). lin-14 regulates the timing of synaptic remodelling in Caenorhabditis elegans.. Nature.

[R6] Kumar J, Choudhary BC, Metpally R, Zheng Q, Nonet ML, Ramanathan S, Klopfenstein DR, Koushika SP (2010). The Caenorhabditis elegans Kinesin-3 motor UNC-104/KIF1A is degraded upon loss of specific binding to cargo.. PLoS Genet.

[R7] Otsuka AJ, Jeyaprakash A, García-Añoveros J, Tang LZ, Fisk G, Hartshorne T, Franco R, Born T (1991). The C. elegans unc-104 gene encodes a putative kinesin heavy chain-like protein.. Neuron.

[R8] Paix A, Folkmann A, Seydoux G (2017). Precision genome editing using CRISPR-Cas9 and linear repair templates in C. elegans.. Methods.

[R9] Sakaguchi-Nakashima A, Meir JY, Jin Y, Matsumoto K, Hisamoto N (2007). LRK-1, a C. elegans PARK8-related kinase, regulates axonal-dendritic polarity of SV proteins.. Curr Biol.

